# Five Canalled and Three-Rooted Primary Second Mandibular Molar

**DOI:** 10.1155/2014/216491

**Published:** 2014-07-24

**Authors:** Haridoss Selvakumar, Swaminathan Kavitha, Rajendran Bharathan, Jacob Sam Varghese

**Affiliations:** ^1^Department of Pedodontics, SRM Dental College, SRM University, AG1 Guru Royal Palace, Rayala Nagar 1st Main Road, Ramapuram, Chennai 600089, India; ^2^Department of Pedodontics, Faculty of Dental Sciences, Sri Ramachandra University, Chennai 600116, India; ^3^Department of Pedodontics, Sri Ramakrishna Dental College and Hospital, Coimbatore 641006, India; ^4^Department of Paedodontia, Dr. Sunny Medical Centre, Shahba, Sharjah, UAE

## Abstract

A thorough knowledge of root canal anatomy and its variation is necessary for successful completion of root canal procedures. Morphological variations such as additional root canals in human deciduous dentition are rare. A mandibular second primary molar with more than four canals is an interesting example of anatomic variations, especially when three of these canals are located in the distal root. This case shows a rare anatomic configuration and points out the importance of looking for additional canals.

## 1. Introduction

Primary multirooted teeth show a greater degree of interconnecting branches between pulp canals and the pulp [1]. The success in root canal procedures is based on understanding the root canal system and its variations by comprehensive cleaning, shaping, and obturation of all root canals. Primary mandibular second molars usually have 2 roots and 3 root canals, with the formation of accessory roots being uncommon [2]. The prevalence of dental anomalies is lower in deciduous dentition than permanent dentition [3]. Tratman (1938) found 3 rooted mandibular molars were rare (frequency < 1%) in the primary dentition [4]. Accessory roots in primary mandibular molars, especially in second molars, were reported amongst Danish, Japanese, Chinese, Taiwanese, and Korean population groups [5].

The overall prevalence of 3 rooted primary mandibular second molars in a Taiwanese population was 10% [6]. Continuous deposition and resorption of secondary dentin may contribute to altered number and shape of the root canals [7]. Additional root canals may be found radiographically but more often are detected only through clinical investigation of the pulp floor and the pulp chamber. The present paper describes a case of primary mandibular second molar with a canal configuration rarely reported in the literature. The tooth had three roots with 5 root canals (2 mesial canals and 3 root canals on two distal roots). This paper may intensify the complexity of primary mandibular molar variation and is intended to emphasize clinician's awareness of the rare morphology of root canals.

## 2. Case Report

**Figure 1:**
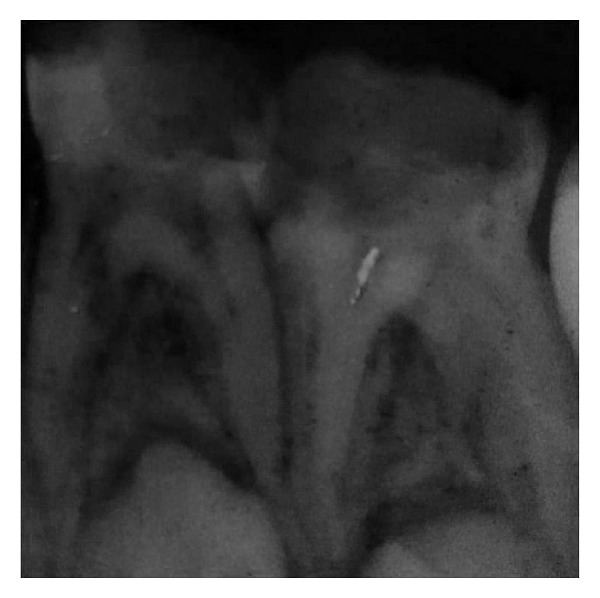
Preoperative radiograph showing advanced dental caries in left mandibular first and second molars.

A 5-year old female patient reported with the chief complaint of pain in the lower left posterior tooth region for the past three days. Pain was spontaneous and aggravated in the night. Clinical examination indicated grossly carious tooth 75. The patient had Frankel behaviour with definitely negative rating. No relevant medical history was given. Radiographic examination of the tooth showed deep caries involving enamel and dentine and extending to the pulp in 75, with a complex root anatomy (Figure 1). From the clinical and radiographic findings, a diagnosis of symptomatic irreversible pulpitis was made for the tooth 75, and a pulpectomy was scheduled. The inferior alveolar nerve block was given with 2% lignocaine containing 1 : 80000 adrenaline (Lignox 2%; Indoco Remedies Ltd., Mumbai, India). The tooth was isolated with a dental dam, and following caries removal of the access cavity was prepared in 75.

**Figure 2:**
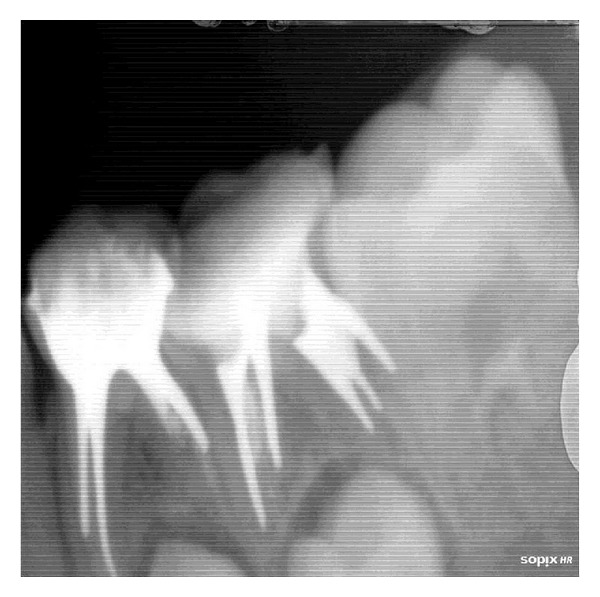
Canals obturated with Metapex paste in 74 and 75 (RVG image).

**Figure 3:**
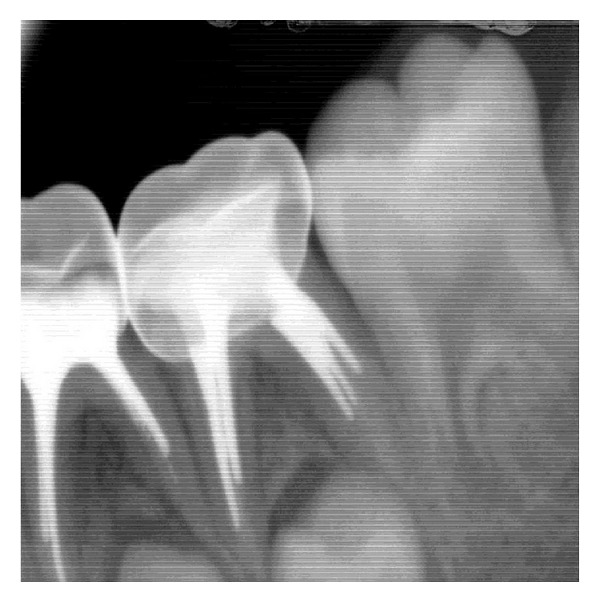
Stainless steel crown in 74 and 75 (RVG image).

All pulp tissue was removed and when the floor of the pulp chamber was reached, three distant canal orifices were initially identified. Canal exploration with a no. 10 file disclosed an additional canal that was located in the distal root midway between the distobuccal and distolingual root canals. Instrumentation was performed in all the canals using H-file (MANI, INC, Japan) and the canals were enlarged to a size 35 using hand instruments. Normal saline irrigation was done throughout the instrumentation. The canals were dried with absorbent paper points (DENTSPLY Tulsa dental specialties, Tulsa, USA) and obturated with Metapex (Meta Biomed Co. Ltd., Korea) using compaction technique. The access was sealed with Glass ionomer Cement (GC Corporation, Tokyo, Japan) and a postoperative periapical radiograph was taken after obturation (Figure 2). After 1 week, stainless steel crown (3M ESPE Unitek, USA) was done and a periapical radiograph was taken (Figure 3). The patient was advised to seek a periodic review every 3 months.

## 3. Discussion

The anatomy of teeth is not always normal. A great number of variations occur in formation, number of roots, and shape of the roots [8]. Routine intraoral radiographs with different angulations help in detecting the presence of extra roots.

Knowledge of anatomic abnormality will also help decrease the failure rate of root canal procedures. There have been several studies on variations in root canal morphology of primary second mandibular molars. Mann et al. reported a 5-year-old child who presented with three-rooted primary mandibular molar [9].

The occurrence of an extra distal root in primary second molar is considered a racial characteristic of certain Indian and Mongoloid populations [10]. Zoremchhingi et al. (2005) using computed tomography evaluated 15 primary second molars and they found one tooth having 3 canals in distal root [11]. Sarkar and Rao (2002) in their ex vivo study found 7.1% with 3 distal root canals in primary second mandibular molars [12]. Rana et al. (2011) reported a case with five root canals in grossly decayed primary second mandibular molar which was extracted and he observed 3 roots with 5 canals (3 mesial and 2 distal) with congenitally bilateral missing of mandibular permanent second premolar (35 and 45) tooth bud [13].

**Figure 4:**
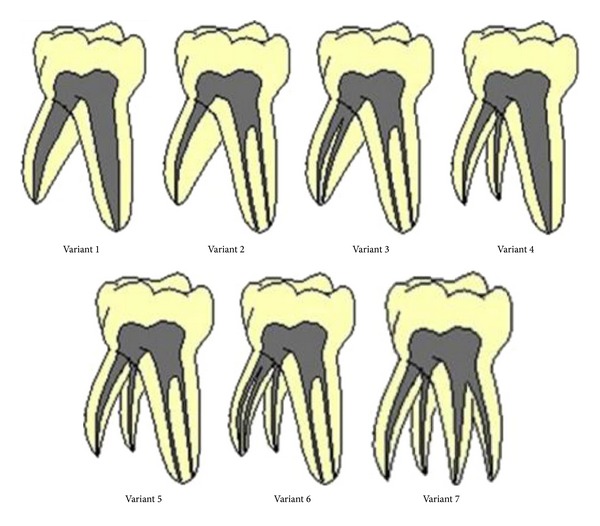
Categorization of the seven variants in primary mandibular second molars by Yang et al. [14].

Yang et al. (2013) evaluated 487 second mandibular molars using CBCT observed seven categories of variants in the root canal anatomy of primary mandibular second molars [14] (Figure 4).

Based on this classification, the case presented here could be considered as Variant 6. Yang et al. (2013) reported one tooth sample had one mesial root and two distal roots and one or two canals in the distobuccal root and one canal in distolingual root. The morphology of this was similar to the present case. The incidence of the variation 6 is low; paediatric dentists should pay attention when performing clinical procedures [14].

The rarity of reports of anomalous root patterns in primary teeth may be more apparent than real. This is because there is only a limited time between the formation and resorption when radiography may indicate their presence and in many cases where primary teeth are extracted the anomalous root pattern is not evident due to root resorption that had taken place [15].

## 4. Conclusion

The knowledge of anatomic characteristics and their possible variation is essential. Examination of clear radiographs taken from different angles and careful evaluation of the internal anatomy of teeth are essential for successful treatment. Knowledge of unfamiliar variations like the case discussed is important as a nontreatment of one additional root or root canal can lead to failure of root canal procedures.
